# Association of the Recurrent *ATP1A1* Variant p.Gly549Arg With Intermediate CMT and Loss of Na,K-ATPase Function

**DOI:** 10.1212/NXG.0000000000200309

**Published:** 2025-09-30

**Authors:** Kerri Spontarelli Fruit, J. Fernando Olivera, Nicolas Colmano, Shawn J. Bird, Brett A. McCray, Sho T. Yano, Steven S. Scherer, Pablo Artigas

**Affiliations:** 1 Department of Cell Physiology and Molecular Biophysics, Center for Membrane Protein Research, Texas Tech University Health Sciences Center, Lubbock;; 2 Department of Neurology, Perelman School of Medicine at the University of Pennsylvania, Philadelphia;; 3 Department of Neurology, University of Michigan Medical School, Ann Arbor;; 4 Section of Pediatric Neurology, Department of Pediatrics, University of Chicago, IL.

## Abstract

**Background and Objectives:**

Charcot-Marie-Tooth (CMT) disease comprises a group of inherited peripheral neuropathies caused by pathogenic variants in various genes, including *ATP1A1*. This gene encodes the ubiquitous α1 subunit of the sodium pump that generates the Na^+^ and K^+^ gradients that are essential for neuronal survival and excitability. We present the clinical cases of 2 unrelated patients with the same *ATP1A1* variant causing dominant intermediate CMT disease and the functional characterization of the variant in the heterologous expression system.

**Methods:**

The patients were evaluated by clinical EMG and by whole-exome sequencing. The function of sodium pump variants was studied with voltage clamp electrophysiology or using ouabain survival curves after heterologous expression in *Xenopus* oocytes or HEK293 cells, respectively. Localization of the variants was evaluated by fluorescence microscopy of HEK293 cells expressing fluorescently tagged sodium pumps.

**Results:**

We describe the cases of 2 unrelated patients who presented in their second decade with a length-dependent and slowly progressive intermediate neuropathy with both axonal and demyelinating features. Whole-exome sequencing identified a de novo c.1645G>A heterozygous variant in *ATP1A1* (p.Gly549Arg) in both patients. The pathogenic nature of the variant was tested through a detailed evaluation of the functional consequences of the Gly549Arg substitution using 2 heterologous expression systems and functional assays that included survival curves of transfected cells and electrophysiology. Patch clamp and 2-electrode voltage clamp electrophysiology experiments showed that the Gly549Arg variant reduced NKA function (≥50%), mainly due to a lower NKA density at the plasma membrane and, to a lesser extent, due to a reduced apparent affinity for intracellular Na^+^. The reduced plasma membrane density was also observed in HEK293 cells simultaneously expressing wildtype and Gly549Arg variants, marked with fluorescent proteins of different colors, suggesting that the mutant may be partially retained in intracellular membranes. No clear dominant-negative effects were identified in these experimental systems.

**Discussion:**

Our results demonstrate that the pathogenic nature of this variant causes considerable loss of function due to diminished plasma membrane localization and kinetic impairments on the enzyme, without obvious dominant-negative effects. Our findings are similar to those previously reported for other CMT disease–causing ATP1A1 variants.

## Introduction

The Na^+^,K^+^-ATPase (NKA, sodium pump) is a membrane protein that spends ATP to build electrochemical gradients for Na^+^ and K^+^, which are essential for excitability and homeostasis.^1-3^ It is an obligatory heterodimer composed of a catalytic α subunit, which contains all the machinery for ATP hydrolysis and ion transport, and an auxiliary β subunit, which is essential for plasmalemma localization. A regulatory FXYD subunit is often found associated with the αβ complex, forming a trimer.^4^ Various neurologic disorders have been associated with germline variants of the genes coding for the α1, α2, and α3 subunits, *ATP1A1*, *ATP1A2*, and *ATP1A3*, respectively. Variants in *ATP1A1* (the focus of this article) are known to cause Charcot-Marie-Tooth (CMT) neuropathies, complex spastic paraplegia, complex developmental syndrome, or hypomagnesemia with seizures and cognitive delay.^5^

CMT is the usual term for inherited neuropathies that are not part of larger syndromes. It presents 3 patterns of nerve conduction abnormalities: the demyelinating type (dominant CMT1, recessive CMT4, CMTX-linked), the axonal type (CMT2, with reduced compound muscle action potential [CMAP] amplitudes), and the intermediate CMTDI, with characteristics of both (reduced conduction velocity and reduced CMAP amplitudes). There are more than 100 different genetic causes of CMT disease.^6^ *ATP1A1-*related CMT disease typically has juvenile-to-adult onset and, like most forms of CMT disease, is characterized by progressive distally accentuated muscle weakness and atrophy, sensory loss, and areflexia. Most described cases primarily involve axonal loss classified as CMT2DD, although affected patients show a wide range of disease onset with incomplete penetrance^7^ and some patients have recently been reported with intermediate characteristics.^8,9^ A nonoverlapping set of *ATP1A1* variants causes a more severe, early-onset neurodevelopmental syndrome,^10-12^ complex spastic paraplegia,^13^ and hypomagnesemia with seizures and cognitive impairment,^14,15^ in which neuropathy is poorly documented (likely due to the earlier onset of the neurodevelopmental phenotype in childhood). Although the selective involvement of peripheral nerves is consistent with the expression of the α1 subunit in motor and sensory neurons as well as myelinating Schwann cells,^7^ given that this protein is expressed in most cell types,^4^ it is perplexing that variants associated with CMT disease cause exclusively peripheral neuropathy.

In this study, we report the neurologic characteristics of 2 unrelated individuals who presented in the clinic with intermediate CMT disease caused by the same de novo *ATP1A1* variant, NM_000701.8:c.1645G>A, corresponding to the Gly549Arg residue substitution in the amino terminus of the α1 protein. To verify pathogenicity and uncover possible pathophysiologic mechanisms of this variant, we performed a detailed evaluation of the functional properties of this previously uncharacterized variant using electrophysiology in *Xenopus* oocytes as well as functional and protein localization studies in human embryonic kidney (HEK293) cells. These studies demonstrate that Gly549Arg causes loss of NKA function that appears to reflect both a large reduction in surface expression and a smaller reduction in the turnover rate of the variant protein, because of a reduced affinity for intracellular Na^+^.

## Methods

### Clinical Studies

Patients underwent evaluation, including clinical electrophysiology, at the University of Pennsylvania and Johns Hopkins University. Whole-exome sequencing was performed by GeneDx.

### Molecular Biology and Heterologous Expression

The Gly549Arg substitution was introduced by PCR mutagenesis into cDNA encoding the human sodium pump α1 subunit and confirmed by sequencing, as previously described.^16,17^ For most experiments, the human cDNA used as a template for the variant also had the double substitution Q118R/N129D, which reduces the affinity of the sodium pump for ouabain, mimicking the naturally ouabain-resistant rodent α1,^18^ allowing separation of the signals from endogenous pumps in the heterologous expression system. For expression in *Xenopus* oocytes, the sequences of the α1 and β1 subunits were cloned into the pSD5 vector and linearized with NdeI (for α1) or BglII (for β1), and cRNA was transcribed in vitro using the SP6 mMessage machine kit (Invitrogen).^16^ Enzymatically dissociated oocytes were purchased from Xenopus1, injected with an equimolar mixture of α1 (75 ng) and β1 (25 ng), and kept in SOS media (100 mM NaCl, 1 mM MgCl_2_, 2 mM KCl, 1.8 mM CaCl_2_, 5 mM HEPES, 2.5 mM pyruvic acid, 1x antibiotic-antimycotic (Gibco), and 5% horse serum (Gibco), titrated to pH 7.5 with NaOH) at 16°C until recording, 3–6 days after injection. In experiments involving co-expression of ouabain-sensitive and ouabain-resistant sodium pumps, equal parts of cRNA were injected of the ouabain-resistant (OR) WT-α1 and an ouabain-sensitive (OS) α1, either OS-WT-α1 (control) or OS-Gly549Arg-α1 (i.e., 37.5 ng of the cRNA for each α, with 25 ng β).

#### Localization Studies

HEK293 cells were transfected with human α1 and β1 constructs, as previously described,^19^ using clones where the α1 subunit coding sequence is preceded by the YFP-coding or the mCerulean-coding sequences connecting to the N-terminus with a 10-residue linker. This strategy produces functional sodium pumps with the rat α1 subunit, as demonstrated by selection of stably transfected CFP-rat α1 HEK293 cell line by culturing in 10 μM ouabain.^20^ This stably transfected cell line (a gift from Seth L. Robia) was used to express YFP-tagged human α1 pumps. Because mCerulean and CFP have identical excitation/emission wavelengths, we refer to both as CFP.

HEK293 cells were grown at 37°C in a humidified incubator (Thermo Scientific) with 5% CO_2_, in Dulbecco's modified Eagle medium with 10% fetal bovine serum and 2% penicillin/streptomycin (all from Gibco). For stably transfected CFP-rat α1 cells, the medium was supplemented with 10 μM ouabain. At 60–80% confluency, the cells were transiently transfected using Polyjet (SignaGen), with the plasmids containing the cDNAs for the α and β subunits (3:1 mass ratio). Forty-three hours after transfection, the cells were lifted using trypsin-EDTA (Gibco), re-plated on poly-d-lysine–coated coverslips, and cultured for another 5 hours before fixation using 4% paraformaldehyde (MP Biomedicals) in phosphate-buffered saline (PBS). Coverslips were mounted with ProLong Diamond Antifade Mountant.

#### Ouabain Survival Assay

HEK293 cells transfected with ouabain-resistant *ATP1A1* expression constructs were passaged 2 days after transfection. Half of the cells were plated in a medium with 10 μM ouabain, and half without. After another 2 days, dead cells were removed by washing them once with PBS and survivors were counted using a cell counter (DeNovix CellDrop).

### Electrophysiology

Electrophysiologic procedures were performed as described.^21^ For the 2-electrode voltage clamp, we used an OC-725C amplifier (Warner Instruments) connected through a Digidata 1440A, a Minidigi 1B, and pClamp software (all from Molecular Devices) for control and acquisition. Current was recorded intermittently at 10 kHz and continuously at 1 kHz. Three molar KCl-filled microelectrodes had resistances between 0.2 and 1 MΩ. Before recording, the oocytes were incubated for 1 hour in Na^+^-loading solution, containing 90 mM NaOH, 20 mM tetraethylammonium (TEA)-OH, 40 mM HEPES, and 0.2 mM EGTA (pH 7.2 with sulfamic acid, ∼220 mOsm/kg). For most experiments, this solution was supplemented with 10 µM ouabain to inhibit endogenous NKA, except for the experiments in which both ouabain-sensitive and ouabain-resistant NKAs were coexpressed. The recording solution contained 150 mM NaOH, 5 mM BaCl_2_, 1 mM MgCl_2_, 0.5 mM CaCl_2_, and 5 mM HEPES titrated to pH 7.4 with methanesulfonic acid (MS, ∼300 mOsm/kg). K^+^_o_ was added by mixing the Na^+^_o_ solution with an external solution where 150 mM KOH replaced NaOH.

For the giant inside-out patch clamp, we used an Axopatch 200B amplifier, a Digidata 1550A A/D converter, a Minidigi 1B, and pClamp software (all from Molecular Devices). The current was recorded continuously at 1 kHz. Fire-polished borosilicate glass pipettes (∼15–20-um diameter, 0.5–0.7-MΩ resistance) were filled with a solution composed of 135 mM NMDG^+^, 5 mM KCl, 5 mM BaCl_2_, 1 mM MgCl_2_, 0.5 mM CaCl_2_, 5 mM HEPES, and 1 µM ouabain (pH 7.4 with HCl). The patch was formed in a solution with 100 mM KOH, 20 mM KCl, 10 mM MgCl_2_, 2 mM EGTA, and 10 mM HEPES (pH 7.0 with l-aspartic acid). After excision, the patches were perfused with intracellular solutions made by mixing K^+^ or Na^+^ solutions containing, respectively, 140 mM KOH or 140 mM NaOH, 10 mM TEA-Cl, 5 mM EGTA, 5 mM HEPES, and 1 mM MgCl_2_ (pH 7.4 with glutamic acid). NKA was activated in the presence of the indicated Na^+^ concentration, by adding MgATP to the bath solution, from a stock solution containing either 163 or 200 mM MgATP (pH 7 with NMDG^+^) stored at −20 °C. All recording solutions were designed to mimic the cationic composition and osmolarity of mammalian physiologic solutions. Ouabain was dissolved directly in external solutions.

### Imaging

Cells were imaged using a 40x oil-immersion objective on a Nikon T1-E confocal microscope. Excitation-laser wavelengths were 445 nm for CFP and 514 nm for YFP. Images were acquired at the emission wavelengths 485 ± 35 and 545 ± 30 nm, respectively.

### Analysis

Data analysis was performed with Clampfit (Molecular Devices) and Origin (OriginLab). Dose-response curves for pump-current activation by K^+^_o_ or Na^+^_i_ were fitted with a Hill equation ($I=I_{0}+I_{\max}\left[\frac{{\left[S\right]}^{n}}{K_{0.5}^{n}+{\left[S\right]}^{n}}\right]$ (where I_max_ is the current at infinite substrate (S), I_0_ is the current at zero substrate, *n* is the Hill coefficient, and K_0.5_ is the [S] that causes half-maximal activation).

For transient charge movement, currents at 10 mM ouabain were subtracted from those elicited by the same pulse protocol in the absence of ouabain. The transient ouabain-sensitive currents were baseline-corrected and integrated to obtain the charge (Q) moved during the pulse at each voltage V (V). The *Q-V curve* was fitted with a Boltzmann distribution:

$$Q=Q_{\text{hyp}}-\frac{Q_{\text{tot}}}{1+\exp\left(\frac{z_{q}e\left(V-V_{\frac{1}{2}}\right)}{\text{kT}}\right)}$$

where Q_hyp_ is the charge moved by hyperpolarizing pulses, Q_tot_ is the total charge moved, V_1/2_ is the voltage at the center of the curve, and kT/z_q_e is the slope factor that indicates the steepness of the curve (in which z_q_ is the apparent valence of a charge crossing the whole electric field, *e* is the elementary charge, k is the Boltzmann constant, and T is the absolute temperature). Sodium pump current and Q_tot_ were normalized to the average values measured in wildtype (WT)-injected oocytes to account for variability in normal expression levels between oocyte batches.

Plasma membrane (PM) localization of fluorescent signals was performed in isolated cells using Fiji image analysis software.^22^ The 2 manually defined regions of interest (ROIs) were the external cell perimeter and the cytosolic perimeter. The PM area and intensity was determined by subtraction of the 2 ROIs. The intensity per unit area was used to obtain the PM/cytosolic intensity. The same ROIs were applied to YFP and CFP channels.

Throughout the article, graphical error bars are SEM while the averages indicated in the text are accompanied by the SD.

### Standard Protocol Approvals, Registrations, and Patient Consents

This article does not include human or animal experimentation. Patients were identified through commercial laboratory testing, and no pictures or videos are included. Xenopus oocytes were purchased from Xenopus1.

### Data Availability

Anonymized data not published within this article will be made available by request from any qualified investigator.

## Results

### Clinical Characteristics

Patient 1 developed CMT symptoms during early childhood (“prancing-like gait” and falls) but was able to play lacrosse and participate in Irish step dancing between ages 5 and 9. She was neurologically evaluated at age 14 for increasing tripping and falls. Her clinical examination showed distal weakness in the arms and legs, absent ankle reflexes, mildly reduced vibration sensation in the toes, and normal pinprick sensation and proprioception (Table 1). Nerve conduction studies (NCS) showed reduced CMAP amplitudes and mildly slowed conduction velocities (Table 2), and needle EMG showed moderate chronic denervation in a distal muscle (tibialis anterior). Her progenitors and siblings were unaffected. Initial genetic tests for *PMP22*, *GJB1*, *MPZ*, *EGR2*, *NEFL*, *PRX*, *GDAP1*, *SIMPLE*, and *MFN2* were normal. Ankle-foot orthoses (AFOs) were prescribed.

**Table 1:** Clinical Summary

	Patient 1				Patient 2	
	14 y	22 y	28 y	31 y	26 y	31 y
Finger extensors	4		4+	4+		4+
FDI	4	4	4	4	4	3
APB	4	4	4	4	4	3
Ankle dorsiflexion	3	4- (L), 2 (R)	2	3 (L), <2 (R)	3	3
Ankle plantar flexion	5	5	5	5	3	4-(L), 4(R)
Vibration (Rydell-Seiffer tuning fork)	Normal to mildly decreased	0 toes 3 ankles 5 knees	0 toes 3 ankles 5 knees	0 toes 0 ankles 3 knees		0 toes 0 ankles 1 knees
Pinprick	Normal	Reduced to above ankles	Reduced to above ankles	Reduced to lower calves	Mildly reduced distally	Normal
Reflexes	Absent at ankles		Absent at ankles and knees	Absent at ankles and knees	Absent at ankles	Absent at ankles

Abbreviations: (L) = left; (R) = right; APB = abductor pollicis brevis; FDI = first dorsal interosseous muscle.

The clinical characteristics of the patients regarding strength, sensation, and reflexes in the extremities. Strength is recorded with the Medical Research Council scale (0–5).

**Table 2:** Summary of Nerve Conduction Studies

Patient #(age in years)	Motor responses (left/right): CMAP amplitude in mV; (conduction velocity in m/s)				Sensory responses (left/right): SNAP amplitude in μV; (conduction velocity in m/s)			
	Ulnar (ADM)	Median (APB)	Peroneal (EDB)	Tibial (AH)	Median (O)	Ulnar (O)	Radial (A)	Sural (A)
Patient 1 (14)	−/−	−/3.8 (35)	−/0.1 (25)	−/−	−/−	−/−	−/9.7 (34)	−/5.7
Patient 1 (22)	−/8.4 (45)	−/3.1 (42)	−/NR	−/1.7	−/NR	−/4.4 (41)	−/3.5 (44)	−/NR
Patient 1 (29)	8.3 (50)/−	3.9 (40)/−	0.2 (23)/−	0.5 (30)/−	NR/−	NR/−	NR/−	NR/−
Patient 2 (26)	6.2 (51)/6.2 (51)	1.2 (35)/2.0 (38)	−/NR	−/−	−/NR	−/NR	−/NR	−/NR

Abbreviations: (O) = orthodromic; (A) antidromic; - = not evaluated; ADM = adductor digiti minimi; AH = abductor hallucis; APB = abductor pollicis brevis; CMAP = compound muscle action potential; EDB = extensor digitorum brevis; NR = no response; SNAP = sensory nerve action potential.

Normal values: ulnar CMAP at ADM: ≥6 mV; median CMAP at APB: ≥6 mV; peroneal CMAP at EDB: ≥2 mV; tibial CMAP at AH ≥ 4 mV; median SNAP: ≥10 μV; ulnar SNAP: ≥7 μV; radial SNAP: ≥15 μV; sural SNAP: ≥7 μV.

Her symptoms progressed year by year, and she had tendon transfers on both feet (at ages 17 and 18), which improved her foot drop and gait but were complicated by a burning sensation on the bottoms of her feet postoperatively. At age 22, she had no symptoms in her hands but there was weakness in the intrinsic hand muscles and ankle dorsiflexion. Ankle plantar flexion was normal, and she could stand on her toes 1 foot at a time. Vibration and pinprick were reduced in a length-dependent pattern. Whole-exome sequencing uncovered a c.1645G>A variant in *ATP1A1* (p.Gly549Arg), which was absent in both parents and was not present in gnomAD. Her examination was unchanged at age 29, but her vibration sensation worsened at age 31 (Table 1). Repeat NCS demonstrated a severe axonal neuropathy affecting sensory and motor axons with little progression between ages 22 and 29. There was length-dependent chronic denervation in the left arm that was severe in distal muscles (Table 2).

Patient 2 had normal development and was sufficiently athletic to play softball in high school. She reported tripping since age 16, ankle sprains in high school, and using AFOs since age 26. She had numbness and mild burning pain in her feet since age 24 and numbness in her hands since age 26. Her parents and siblings were unaffected. At age 26, NCS showed a moderate-to-severe neuropathy with conduction slowing that was particularly pronounced in median motor responses (Table 2). Needle EMG showed chronic denervation that was moderate in the thighs and severe in the calves. Workup for possible acquired inflammatory demyelinating neuropathy was negative, including negative or normal t serum protein electrophoresis with immunofixation, serum vascular endothelial growth factor level, anti-myelin–associated glycoprotein antibody, skeletal survey, and CSF protein. CMT was thought to be the most likely diagnosis, but a 53-gene panel failed to reveal pathogenic variants. Treatment with IV immunoglobulins for 1.5 years improved her fatigue but not her weakness or other symptoms.

Clinical and electrophysiologic evaluation remained unchanged at age 29. There was no clinical improvement with 3 months of i.v. corticosteroids. At age 30, whole-exome sequencing revealed a c.1645G>A (p.Gly549Arg) variant in *ATP1A1* that was absent in both parents. Her examination at age 31 showed a slight worsening of symptoms compared with that at age 26 (Table 1).

### Gly549Arg Causes Loss of NKA Function

**Figure 1:**
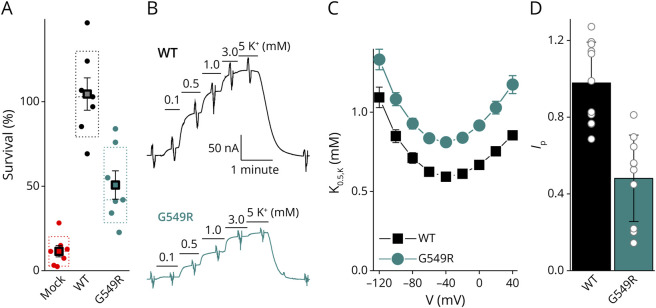
Gly549Arg (G549R) Causes Loss of NKA Function (A) HEK293 cells transfected with the indicated ouabain-resistant *ATP1A1* expression constructs or mock-transfected cells (Mock) were challenged with 10 µM ouabain for 2 days. However, WT constructs supported cell survival and G549R partially but significantly (*t* test, *p* < 0.002) decreased cell survival, indicating impaired NKA function. (B) Representative current recordings from Na^+^-loaded oocytes clamped at −50 mV and expressing WT-α1β1 (*top*) or G549R-α1β1 (*bottom*) in external solution containing Na^+^ and the indicated [K^+^_o_]. K^+^_o_-induced outward pump current in a concentration-dependent manner. The ramp-like deflections in the trace indicate application of 100 ms-long voltage pulses to measure the voltage dependence of I_P_ at each ion concentration, to obtain the K_0.5_. (C) Mean K_0.5_-V from Hill fits to the K^+^_o_ dependence of *I*_P_. Error bars are SD. The *t* test indicates that the 2 means are significantly different (*t* test, *p* < 0.0001). (D) Mean 4.5 mM K^+^_o_-induced current at −50 mV, normalized to the average from WT-injected oocytes measured on the same day.

Based on the American College of Medical Genetics criteria, we classified the Gly549Arg variant as likely pathogenic,^23^ applying PM2_Supporting (absent from the gnomAD population database), PS2_Moderate (*de novo* in 2 probands with a genetically heterogeneous phenotype), PP3_Strong (strongly predicted pathogenic with score 0.947 by the REVEL algorithm), and PP2 (*ATP1A1* is highly missense-intolerant, Z = 8.24, compared with other genes). To confirm pathogenicity of the variant, we performed a series of in vitro assays. The overall function of Gly549Arg was first evaluated using an ouabain survival assay (Figure 1). HEK293 cells have endogenous sodium pumps highly sensitive to the specific inhibitor ouabain (IC_50_ < 100 nM^24^) and, therefore, die in the presence of low micromolar concentrations of the toxin. Ouabain toxicity is reduced in transfected cells that express functional ouabain-resistant sodium pumps (IC_50_ < 100 μM, in Methods). Survival of Gly549Arg-transfected cells was significantly higher than mock-transfected cells and significantly lower than WT-transfected cells (*p* < 0.002, Figure 1A), suggesting partial loss of function.

We then evaluated the effects of Gly549Arg on enzymatic function using electrophysiology. Representative experiments using 2 oocytes held at −50 mV (Figure 1B), expressing either WT *(top*) or Gly549Arg-α1 (*bottom*), illustrate the concentration-dependent activation by extracellular K^+^ (K^+^_o_). Gly549Arg slightly increased K_0.5,K_ at all voltages (Figure 1C) but halved the current amplitude at physiologic K^+^_o_ (Figure 1D).

**Figure 2:**
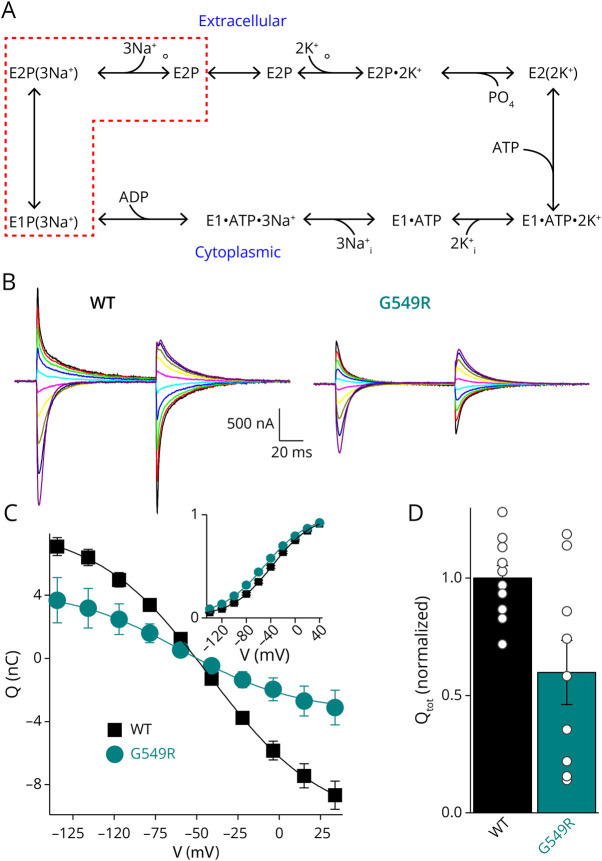
Transient Charge Movement (A) The Post-Albers kinetic scheme describing NKA function. When voltage pulses are applied in the absence of K^+^, the pumps shuttle back and forth between the states enclosed by the red dashed box, causing transient currents as the ions move within the protein's electric field. (B) Representative ouabain-sensitive currents (current before ouabain − current after ouabain) elicited by voltages pulses between −140 and +40 mV (40-mV increments shown) from oocytes injected with WT (*left*) or Gly549Arg (G549R, *right*). (C) Mean charge − voltage (Q-V) for WT and G549R oocytes fitted with a Boltzmann distribution (line plot, see Methods). The inset shows the normalized Q-V to illustrate the leftward shift of the G549R curve (WT V_0.5_ = −37 mV; G549R V_0.5_ = −50 mV). (D) Mean Q_tot_ normalized to average value of WT on the same day. Error bars represent SEM. Data from 5 batches with at least 2 WT-injected oocytes recorded. Mean values are significantly different, *t* test *p* = 0.01.

To test whether the diminished current amplitude was caused by a lower turnover rate or a reduced sodium pump density at the plasma membrane, we measured the ouabain-sensitive transient currents elicited by square voltage pulses obtained in the absence of K^+^_o_ (Figure 2). Under this condition, the pumps are restricted to the voltage-dependent transition between E_1_P(3Na) and E_2_P (dotted red box in the reaction cycle of Figure 2A). The same pulse protocol was repeated before and after application of 10 mM ouabain. The pump-mediated currents (Figure 2B, current without ouabain – current in ouabain) were integrated to obtain the charge moved (Q), which is plotted as a function of the applied voltage (*Q-V*, Figure 2C). The Boltzmann distribution fitted to the curves (line plots) yields the total charge (*Q*_tot_, proportional to the number of functional pumps, but independent of the turnover rate), and the center of the curve (V_0.5_, an indicator of changes in apparent affinity for Na^+^_o_^25^). Gly549Arg-injected oocytes have approximately 50% less total charge than WT-injected ones (Figure 2D). Thus, the reduced sodium pump current in Figure 1 is mainly due to fewer Na pumps in the plasma membrane. The leftward shift in V_1/2_ suggests a small decrease in affinity for Na^+^_o_.

**Figure 3:**
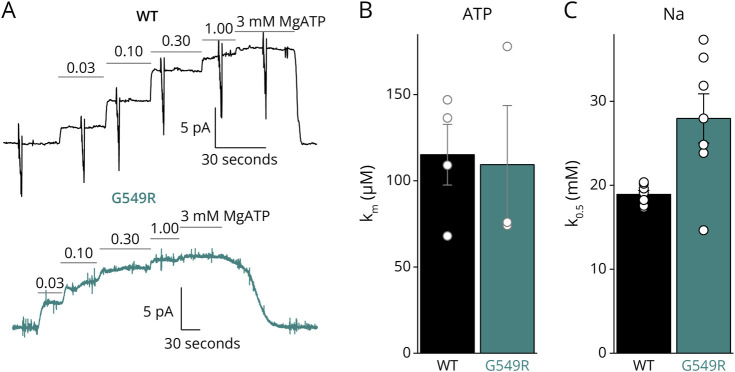
Affinity for Intracellular Ligands (A) ATP-activated current traces from inside-out giant patches excised from oocytes expressing WT-α1β1 (*top*) and G549R-α1β1 (*bottom*), at 0 mV. The current was induced by changes in [MgATP] at 50 mM Na^+^_i_ (90 mM K^+^). The ramp-like deflections in the WT trace absent in the G549R patch correspond to brief 50 ms-long changes in voltage. (B) Bar graph showing the mean K_m,ATP_ overlapped with data from individual experiments. (C) Bar graph with the mean K_0.5,Na_ from Hill fits to dose-response experiments with Na^+^ (where Na^+^ was substituted with K^+^ in the intracellular bath solution Na^+^ + K^+^ = 140 mM). The data from individual patches were fitted globally with shared Hill coefficients *n*_*H*_ = 2.4 ± 0.4 for WT and *n*_*H*_ = 1.65 ± 0.3 for G549R patches (SEM from the global fits to data from 7 patches for each construct). The higher variability in parameters from patches with G549R probably reflects their lower signals (the 50 mM Na^+^ + MgATP induced currents of 7.4 ± 3.6 pA for WT and 4.1 ± 2.4 for G549R patches, SD, n = 10 in both cases) consistent with the lower current amplitudes observed in whole-oocyte recordings.

We used inside-out giant patches to assess changes in affinity for intracellular ligands (Figure 3). Gly549Arg had similar apparent affinity as WT for MgATP activation of pump current (Figure 3A, B), but the K_0.5_ for Na^+^ was significantly reduced (*t* test, *p* < 0.01), from 18.9 ± 1.2 mM (n = 7, SD) in WT to 28.0 ± 7.7 mM (n = 7, SD) in Gly549Arg (Figure 3C). Because Na^+^ binding to its intracellular-facing sites is a highly cooperative process^16,21^ and a rate-limiting step for the function of the sodium pump under physiologic conditions, the increased K_0.5_ may contribute to further loss of function in the patients' neurons.

**Figure 4:**
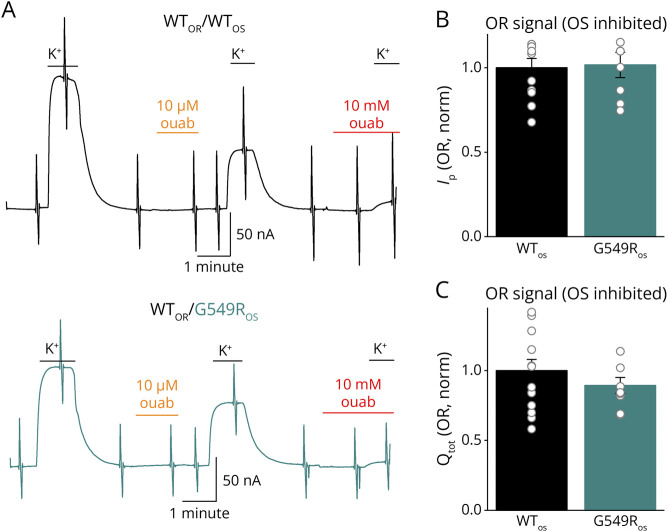
Coexpression of G549R and WT (A) Representative current recordings at −50 mV coexpressing WT_OR_/WT_OS_ (*top*) or WT_OR_/G549R_OS_ (*bottom*). Oocytes were bathed in an extracellular solution of 150 mM Na^+^. The current elicited by the first application of 4.5 mM K^+^ is attributed to both the ouabain-sensitive and ouabain-resistant pump. Subsequent application of 10 μM ouabain inhibited the ouabain-sensitive pumps, leaving the ouabain-resistant WT pumps available for activation during the second application of K^+^. Ten mM ouabain was used to completely inhibit all pumps. (B) Mean pump current (*I*_P_) activated by K^+^ application after adding 10 μM. Data were normalized to the average I_P_ measured in the WT_OR_/WT_OS_ oocytes on the same day. (C) The 10 mM ouabain–inhibited transient currents were obtained at the end of the experiments as those in A (before the last application of K^+^); the Q_tot_ of the ouabain-resistant pumps was calculated from Boltzmann plots and normalized to the WT_OR_/WT_OS_ average measured on the same day.

To address whether the presence of the variant may reduce the activity of coexpressed WT enzymes, as has been shown for *ATP1A3* variants causing alternating hemiplegia of childhood,^26^ we coinjected WT and Gly549Arg together with β1 (Figure 4). Current recordings from representative oocytes expressing either WT ouabain-sensitive (WT_OS_) and WT ouabain-resistant (WT_OR_) pumps or Gly549Arg-OS (G549R_OS_) and WT_OR_ pumps illustrate how the experiment is performed (Figure 4A). The total population of pumps was first activated by application of 4.5 mM K^+^, and after K^+^ withdrawal, 10 μM ouabain was applied to inhibit the ouabain-sensitive NKA fraction. After removal of ouabain, the ouabain-sensitive pumps remained inhibited and the second application of K^+^ activated only WT_OR_ pumps. The ouabain-resistant pump current amplitude (Figure 4B) and the total charge of the ouabain-resistant component (Figure 4C) were normalized to the mean ouabain-resistant amplitudes from WT_OR_ and WT_OS_-injected oocytes. These results indicate that the presence of the variant does not alter the function of the WT type allele.

### Localization of Gly549Arg and WT Within Mammalian Cells

**Figure 5:**
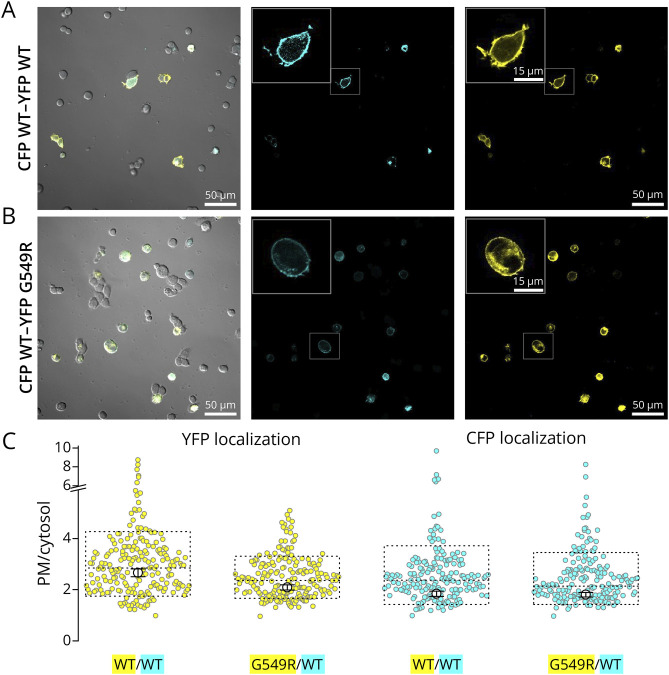
Localization of G549R and WT in the Same Cell (A, B) Representative images from HEK293 cells cotransfected with CFP-tagged and YFP-tagged α1, overlaid with the transmission detector (*left*), CFP signal (*center*), and YFP signal (*right*). CFP-A WT-α1/YFP-WT-α1 cotransfection is shown in (A) and CFP-WT-α1 and YFP-G549R-α1 in (B). The inset shows a zoom-in of the boxed cell. (C) Intensity in the plasma membrane divided by the intensity in the cytosol for ≥200 images of each transfection pair. Data from stable and transiently expressed CFP were pulled together (see Methods). Box indicates SD and median. The mean and SEM are shown in the center.

To study the localization of WT and Gly549Arg in mammalian cells, we simultaneously expressed 2 α1 subunits, N-terminally tagged with fluorescent proteins of different colors (Figure 5). The cells cotransfected with YFP-WT-α1 and CFP-WT-α1 show both signals localizing primarily to the plasma membrane (Figure 5A) while cells with YFP-Gly549Arg-α1 and CFP-WT-α1 show increased cytosolic localization of the YFP signal but not the CFP signal (Figure 5B). We also used a slightly different approach, by transiently transfecting YFP-tagged WT or Gly549Arg α1 on a HEK293 cell line that was previously stably transfected with a CFP-tagged rat WT α1,^20^ and obtained similar results (not shown). The ratio of the intensities/unit area at the plasma membrane and at the cytosol was used to quantify localization (Figure 5C). The ratio of intensities was compared with the Mann-Whitney test; the YFP-G549R variant is significantly less localized to the plasma membrane than the WT (*p* < 0.0001), but the CFP-WT cotransfected with it is not (*p* = 0.13).

## Discussion

We have described the neurologic characteristics of 2 unrelated patients with the same Gly549Arg substitution in the sodium pump α1 subunit (coded by *ATP1A1*). Both patients had a similar phenotype—clinical onset in the second decade with manifestations of a sensory and motor neuropathy, followed by slow progression. Clinical neurophysiology confirmed sensory and motor axonal loss, with “intermediate slowing” (35–45 m/s) of some motor responses in the arms. The phenotype we found in these 2 unrelated patients is similar to that described in previous reports of CMT2DD in other heterozygous carriers of the p.Gly549Arg variant, a 40-year-old Iranian man^27^ as well as a Spanish adolescent and his father,^28^ who presented intermediate conduction velocities. The phenotype of patients carrying p.Gly549Arg resembles those reported in patients with variants p.Ile592Thr, p.Asp597Thr, p.Pro600Thr, p.Pro600Arg, or p.Gly877Ser. With the caveat that CMT2DD-causing variants cause variable phenotypes, the patients with the p.Gly549Arg variant may exhibit a more severe phenotype than those with the p.Leu48Arg variant^7^ and a less severe phenotype than those with the p.Asp601Phe or p.Asp811Ala variants.^7-9^

It is unclear whether *ATP1A1* variants that cause other disease phenotypes, such as hypomagnesemia with seizures and intellectual disability or complex neurodevelopmental delay, may also present with peripheral neuropathies. This is because the very early appearance of the more severe CNS phenotypes may not have allowed enough time for the neuropathic phenotype to develop. One might expect that variants causing a particular set of phenotypes would present certain common characteristics. To date, in vitro studies heterologously expressing the 4 reported mutants that cause hypomagnesemia with seizures and intellectual disability have failed to demonstrate a common mechanism. Although all 4 mutants present certain degree of loss of NKA function,^14,15^ only 3 variants appear to carry passive inward “leak” currents similar to those observed in most *ATP1A1* somatic variants from aldosterone-producing adrenal adenomas, which cause hyperaldosteronism and secondary hypertension.^16,29,30^ Many *ATP1A2*^31^ and ATP1A3^32,33^ variants have also been reported to carry inward currents. Variants causing complex neurodevelopmental delay^12^ or spastic paraplegia^13^ have not been studied in sufficient detail.

Using diverse approaches, we found that Gly549Arg causes substantial loss of function, as demonstrated by the lower ouabain survival in HEK cells expressing the ouabain-resistant Gly549Arg compared with cells expressing the ouabain-resistant WT α1 (Figure 1). The variant's functional impairment appears to reflect 2 primary mechanisms: a trafficking defect resulting in approximately 50% diminished number of pumps at the plasma membrane (Figure 2) and the reduced apparent affinity for intracellular Na^+^ (Figure 3), which would reduce the number of functional pumps at physiologic Na^+^ by a further 30% (under the reasonable assumption that the resting physiologic Na^+^ concentration is equal to the K_0.5_ for WT pumps). Thus, these 2 loss-of-function mechanisms combined would mean that the Gly549Arg variant transports considerably less than the WT allele product, causing a partial loss of total sodium pump function similar to or greater than the effects described for other CMT-causing *ATP1A1* variants.^7,8,34^

Overall, if the WT pumps express and function normally in the presence of the disease variant allele (as suggested by the experiments in Figures 4 and 5), then one would expect that the axons of carrier patients will have a 35%–50% lower sodium pump capacity than unaffected individuals. Such a reduction is expected to be equal to or smaller than the reduction observed in heterozygous *Atp1a1*^+/−^ knockout mice (which have not been reported to develop neuropathy) or in adults, who carried the early truncation variant p.Tyr148* in *ATP1A1* but lacked CMT or other disease characteristics frequently found in patients with other *ATP1A1* variants.^17^ One possible explanation for this conundrum may be that the presence of the malfunctioning protein impedes a plausible compensatory mechanism involving slightly increased expression of the normal *ATP1A1* or *ATP1A3* alleles (small enough to not be detected in Western blots for protein expression in mice, cf. Figure 1in Ref. 17), therefore uncovering haploinsufficiency that requires the presence of the malfunctioning protein product.

An alternative explanation, however, may be that the lack of clear interference from the variant protein on WT function is inherent to the non–nervous system heterologous expression systems where the variants are overexpressed. Both primary motor and sensory neurons (and their axons), as well as myelinating Schwann cells, express *ATP1A1*.^7,35^ The nonuniform expression patterns of the sodium pump isoforms in these cell types are controlled by signals that remain largely unknown. Thus, neuron-specific and Schwann cell–specific dominant-negative effects that alter α1 localization may explain why the pathophysiology of Gly549Arg and other CMT disease–causing *ATP1A1* variants is constrained to the peripheral nervous system, although the mutant α1 isoform is present in nearly every cell of the body. Evaluation of these possibilities will require development and use of more complex cell and animal models of disease.

## Glossary

CMAP: compound muscle action potential
CMT: Charcot-Marie-Tooth
NCS: nerve conduction studies
